# Supplementary material for: Engaging teenagers in improving their health behaviours and increasing their interest in science (Evaluation of LifeLab Southampton): study protocol for a cluster randomized controlled trial
Source: Trials. 2015 Aug 21;16:372. doi: 10.1186/s13063-015-0890-z (PMC4546100; doi:10.1186/s13063-015-0890-z)
Supplement: Additional file 1: — Pre-questionnaire script. (PDF 66 kb) [file 13063_2015_890_MOESM1_ESM.pdf]

LIFELAB

Pre-questionnaire script:

I'm one of the teachers at LifeLab and I will explain what you're going to be doing today and why. LifeLab is part of the University of Southampton and based at Southampton General Hospital. We really want to understand what young people like yourselves know about health, now and in the future, for themselves and for their future children. We all hear in the news everyday about the health problems facing us as we get older. Many of these are linked to our lifestyle choices, for example; heart disease, type 2 diabetes and some kinds of cancer. We want to be able to reduce the risk for young people of developing these diseases as they get older. In order to do that we need to collect data about what young people think and understand about health now. To do this we have approached schools in the South of England and your school has been one of the ones chosen to participate in the LifeLab program.

You should have received a letter explaining our research in more detail. Our study is a randomised control trial. This means that each school is randomly allocated to be either an intervention school or a control school. Intervention schools test out the LifeLab teaching materials and control schools provide important baseline data so that we can compare the two groups. We collect the same data from both groups in the form of an on-line questionnaire. We carry out two questionnaires, one at the start called a pre-questionnaire and one about 12 months later called the post-questionnaire. By completing these questionnaires you are contributing to real science which could make real changes so it's really important to be honest and accurate so that we know our data is valid.

As all good scientists we're not going to look at any individual's response, we will be looking for patterns and trends in the data and comparing any changes. Eventually the questionnaire will become anonymous. However, we need to match your pre and post questionnaires first. To help us do this we will be asking for three sources of information; your name, your postcode and your date of birth. We do this because some of these may change. Some people change their name, some people move. Hopefully your date of birth will remain the same and this will enable us to match your pre and post questionnaire accurately. Don't worry if you can't remember your postcode, your teacher will be able to help you with this.

To access the on-line questionnaire you first need to log on as normal then enter the following website into the web browser: www.isurvey.soton.ac.uk/. Your teacher will have a number to add to the final part of the web address. Make sure you use the web address bar, not the search engine. A copy of the letter will be displayed, that you have been sent home. You may wish to read it again. When you scroll down to the bottom you will find a small tick box that you need to click on to confirm you are happy to continue and complete the questionnaire. Use the green tabs at the bottom of the page to move you on to the next page or go back a page. Don't use the back button at the top as you may log out and will have to start the questionnaire again.

Remember this is not an assessment, there is no right or wrong answer. It is your individual thoughts and opinions that count. Thank you very much for taking part in the LifeLab project.
